# Self-reported and objectively measured physical activity in people with and without chronic heart failure: UK Biobank analysis

**DOI:** 10.1136/openhrt-2019-001099

**Published:** 2020-02-19

**Authors:** Johanna O'Donnell, Karl Smith-Byrne, Carmelo Velardo, Nathalie Conrad, Gholamreza Salimi-Khorshidi, Aiden Doherty, Terence Dwyer, Lionel Tarassenko, Kazem Rahimi

**Affiliations:** 1 George Institute for Global Health, University of Oxford, Oxford, Oxfordshire, UK; 2 Institute of Biomedical Engineering, University of Oxford, Oxford, UK; 3 Nuffield Department of Population Health, University of Oxford, Oxford, UK

**Keywords:** heart failure, epidemiology, heart failure treatment, public health

## Abstract

**Objective:**

The impact of heart failure (HF) on perceived and objectively measured levels of physical activity (PA) can inform risk stratification and treatment recommendation. We aimed to compare self-reported and objectively measured PA levels in a large sample of participants with and without HF.

**Methods:**

A validated PA questionnaire was used to estimate self-reported weekly PA among 1600 participants with HF and 387 580 participants without HF. Accelerometer data were studied in 596 participants with HF and 96 105 participants without HF for a period of 7 days. Using multivariable linear regression models, we compared the PA levels between participants with HF and without HF, focusing on both the average daily PA levels and the intensity of PAs throughout the day.

**Results:**

PA levels were significantly lower in participants with HF using both self-report (excess metabolic equivalent of task hours per week of 26.5 (95% CI 24.7 to 28.4) vs 34.7 (95% CI 34.5 to 34.9), respectively (p<0.001)) and accelerometer measures (mean accelerations of 23.7 milligravity (95% CI 23.1 to 24.4) vs 28.1 milligravity (95% CI 28.0 to 28.1), respectively (p<0.001)). Findings were consistent across different PA intensities. Hour-by-hour comparisons showed that accelerometer-derived PA levels of patients with HF were reduced throughout the day.

**Conclusion:**

Perceived and objectively recorded PA levels of patients with chronic HF are significantly lower than those of individuals without HF. This difference is continuous throughout the different hours of the day, with individuals with HF being on average 16% less active than individuals without HF. In patients with HF, increases in everyday activity may be a potential alternative to structured exercise programmes.

**Key messages**

**What is already known about this subject?**

- Reduced levels of physical activity (PA) in heart failure (HF) patients have been associated with more severe levels of breathlessness and an increased risk of long-term mortality, however rlittle is known about PA levels of HF patients throughout the day and in comparison with non-HF individuals.

**What does this study add?**

- In this study, we compare self-reported and accelerometer-derived PA levels in a large group of individuals with and without HF and show that both perceived and objectively recorded PA levels of patients with chronic HF are significantly lower than those of individuals without HF. In our study, accelerometer-derived measures revealed that PA levels of patients with HF are significantly reduced throughout the day.

**How might this impact on clinical practice?**

- This research allows clinical staff to gain a better understanding of the impact of HF on patients’ day-to-day life. We show that both patients self-reported (perceived) and accelerometer-derived (objective) PA is significantly lower than in participants without HF when adjusting for age and sex. Furthermore, through the added detail provided by accelerometer recordings, we show that PA levels of patients with HF are significantly attenuated throughout the entire day with the exception of night hours, suggesting that HF affects the ability of patients to conduct even basic everyday PA. The latter may also suggest that increases in the activeness during everyday activities may be a potential alternative to a structured physical exercise regimen. Such interventions may be of particular benefit to patients who express concern about potential adverse effects of physical exercise.

## Introduction

In patients with heart failure (HF), increases in levels of physical activity (PA) improve quality of life^1^ and may reduce the risk of hospitalisation and death.^2 3^ Therefore, many national and international guidelines strongly recommend regular PA and structured exercise training for patients with mild to moderate chronic HF.^4–6^ However, these recommendations are rarely achieved by patients.^7^

Factors influencing the adherence to recommended PA guidelines in HF include fluctuating health and patients’ external and internal motivations.^8^ In addition, patients’ concern about the risks associated with overdoing physical exercise has been identified as a barrier to adherence^8^; this might be fuelled by poor patient and doctor awareness of individuals’ ability to conduct PA.

While exercise intolerance is a well-studied symptom of HF, levels of everyday PA are less well understood.^5^ Existing research suggests that reduced PA levels in patients with HF are associated with more severe levels of breathlessness^6 7^ and an increased risk of long-term mortality.^9–11^ However, reliable analysis of self-reported and objectively measured PA among patients with HF and their comparison with individuals without HF is limited.^5 12 13^

This study set out to compare PA behaviour of people with and without HF using both self-reported (perceived) and accelerometer-derived (objective) measures and to investigate objectively recorded PA levels of patients with HF throughout the day.

## Methods

### Study population

This research has been conducted using the UK Biobank Resource^14^ (study ID 16032). Between 2006 and 2010, the UK Biobank recruited approximately 500 000 subjects aged 40–69 years from across the UK and collected a range of self-reported data, physiological measurements and biological samples at baseline.

Information about health-related outcomes (before and after the baseline assessment) were obtained through linkage of the UK Biobank with data from the Office for National Statistics for death and emigration information and the Department of Health’s Hospital Episode Statistics for hospital-admission diagnoses (up until March 2015 for English residents that were either NHS-funded or NHS-treated).

### Self-reported PA

Baseline assessment included information on self-reported PA measured through an adapted version of the International PA questionnaire (IPAQ)^15^ completed on a tablet computer. Patients were asked to state how many days they were engaged in more than 10 minutes of each, walking, moderate PA and vigorous PA in a typical week. Participants were then questioned for how many minutes they were engaged in each of the activities on a typical day.

Self-reported PA data were processed using the method described by Bradbury *et al* 16 based on the IPAQ guidelines.^17^ Walking and moderate and vigorous activities were scored at 2.3, 3.0 and 7.0 excess metabolic equivalents of tasks (METs), respectively.^17^ In order to extract participants’ average excess METs per week, the time spent in each of the activities on a typical day was multiplied by the typical number of days doing the exercise and the respective MET scores. Self-reported average daily physically active minutes of <10 min were recorded as 0. After aggregating over the whole week, self-reported values of ≥1260 min per week (equivalent to an average of 3 hours a day) were truncated at 1260 min according to the IPAQ guidelines.^16^ Self-reported PA levels were reported in excess MET-hours per week. Participants who answered ‘do not know’ or ‘prefer not to answer’ to any of the self-reported PA questions were removed from the analysis.

### Accelerometer-derived PA

Accelerometer data were collected for a subset of approximately 100 000 participants between May 2013 and 2015.^18^ UK Biobank participants were contacted via email and provided with an Axivity AX3 accelerometer (Open Lab, Newcastle, UK) if they agreed to take part in the activity data collection. Participants were told to wear the three-axial accelerometer continuously on their dominant wrist for a period of 7 days. The accelerometer recorded data at a frequency of 100 Hz and an acceleration range of ±8 g.

The raw accelerometer data were calibrated^19^ and wear-time periods were identified using the UK Biobank preprocessing methods described by Doherty **et al*.* 18 Signals with poor wear-times or failure to calibrate were removed from the analysis. Accelerometer-based summary measures included the total mean acceleration over the 7-day measurement period, mean hourly acceleration and time spent within a range of different mean acceleration values as a marker of PA intensities.^18^ The proportion of time spent in sedentary, light, moderate and vigorous PAs was thereby defined as the proportion of time spent in accelerations of ≤25, 26–100, 101–425 and >425 milligravity, respectively.^18 20^

### HF definition

**Figure 1:**
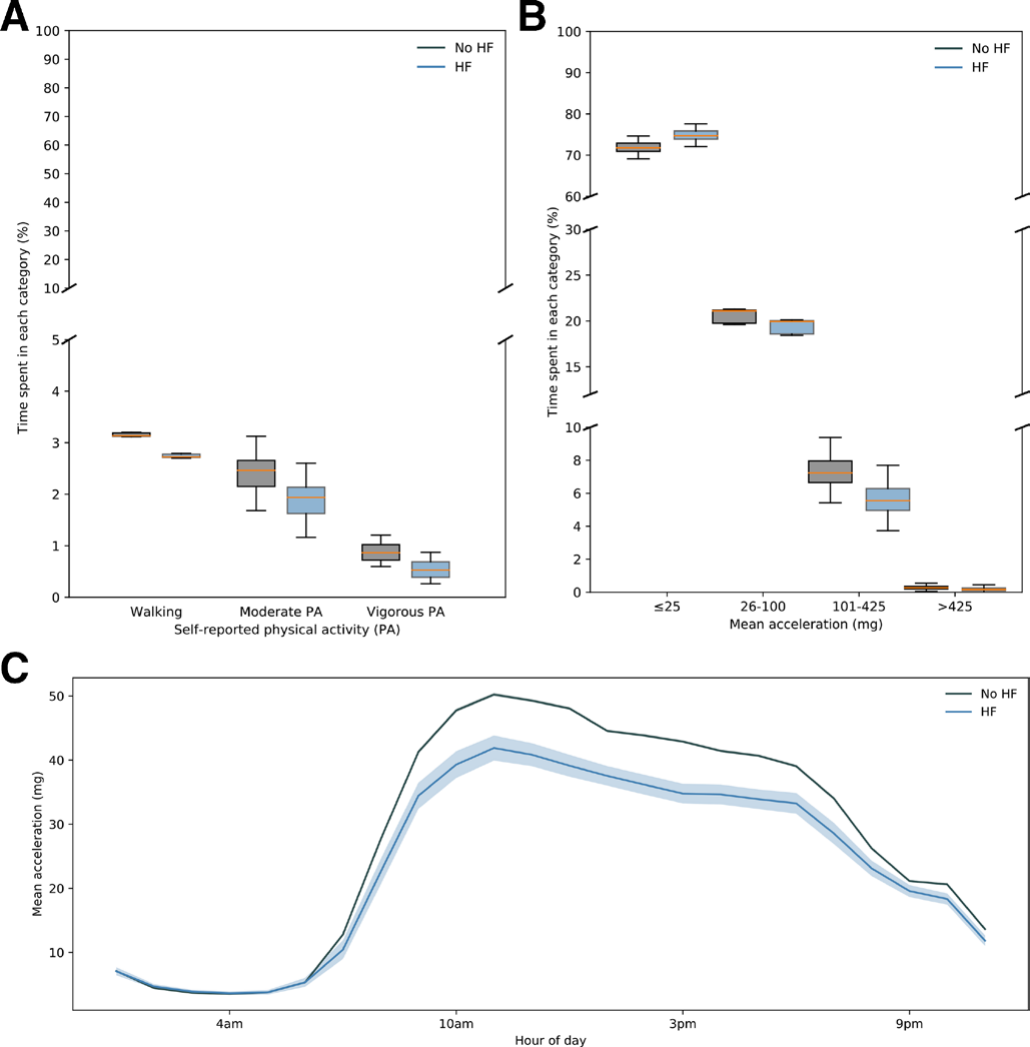
Summary of self-reported and accelerometer-derived PA behaviours in participants with or without HF. (A) Box plots of percentage time spent within different categories of mean age-adjusted/sex-adjusted self-reported PA among participants with (blue) or without (grey) diagnosed HF. All differences are significant (p<0.01). (B) Box plots of percentage time spent within different mean age-adjusted/sex-adjusted acceleration ranges for participants with (blue) and without (grey) diagnosed HF. All differences are significant (p<0.01). (C) Mean (solid line) and CIs (shaded area) of mean acceleration during different hours of the dayfor participants with (blue) and without (grey) diagnosed HF. HF, heart failure; PA, physical activity.

We defined prevalent HF as any primary or secondary discharge diagnosis of HF as indicated by the following codes: ICD10 code 'I500', 'I501' or 'I509' and ICD9 code ‘4280’ or ‘4281’ prior to the assessment of PA. Given that accelerometer data were collected after questionnaire-based PA assessment and were restricted to a subsample of the total cohort, we had two partially overlapping HF populations (figure 1).

### Covariate definitions

Age was defined as age at the time of each PA measurement. All other covariates were based on data collected at baseline. Smoking and alcohol status were divided into never, previous or current. Social deprivation was determined using the Townsend Deprivation Index, which is an area-of-residence-based social deprivation index.^21^

### Statistical analysis

We used multivariable linear regression to compare the mean PA between participants with HF and without HF. Analyses were carried out separately for self-reported and accelerometer-derived PA levels. Primary analyses were adjusted for age and sex. Secondary analyses were additionally adjusted for ethnicity, baseline Body Mass Index (BMI), alcohol status, smoking status and socioeconomic status. We performed two sensitivity analyses to test the impact of timing of HF diagnosis and PA assessment on the observed outcomes. In the first sensitivity analysis, we restricted comparisons to participants who had HF at baseline (ignoring any participants with incident HF report between baseline assessment and accelerometer data collection) to ensure that any observed differences between self-reported and objectively assessed PA levels were not due to a change in population classification over time. In a second sensitivity analysis, we investigated the impact of time since diagnosis of HF on any observed differences in PA levels between HF and non-HF participants. Given that both time since incident diagnosis of HF and time since last admission to hospital with HF could impact PA levels, we selected three time intervals as alternative explanatory variables: time since first report of HF, time since last report of HF hospitalisation where HF was the primary discharge diagnosis and time since last report of HF hospitalisation where HF was the primary or secondary discharge diagnosis. All analyses were done in R.^22^

## Results

Out of a total of 502 602 UK Biobank participants, 389 180 provided valid self-reported PA information at baseline. This included 1600 participants with previously diagnosed HF. Accelerometer-based PA data of sufficient quality (to be included in the analysis) were collected for 96 701 UK Biobank participants between 2013 and 2015, of whom 596 had an HF diagnosis prior to the recording. A subset of these participants (n=244) had HF at baseline; others were diagnosed with HF between the baseline assessment and the recording of accelerometer data. Out of the total of 80 121 with self-reported PA data at baseline and a valid accelerometer recording (overlapping group), 190 had a diagnosis of HF at baseline (see figure 1).

Characteristics of participants in the three subpopulations (self-reported PA, accelerometer-based PA and overlapping PA assessment) by HF status are presented in table 1. Across all populations, patients with HF were on average older than non-HF participants and were more likely to be male. Participants with HF were more likely to be overweight or obese than participants without an HF diagnosis. HF participants were more likely to have given up alcohol and to class themselves as previous or current smokers in all analyses.

**Table 1:** Baseline characteristics of UK Biobank participants

	Self-reported PA			Accelerometer-derived PA			Overlapping PA		
	No HF	HF	P value	No HF	HF	P value	No HF	HF	P value
n	387 580	1600		96 105	596		80 121	190	<0.001
Age (years)	57 (50–63)	63 (59–67)	<0.001	63 (56–69)	66 (65–72)	<0.001	57 (50–62)	62 (58–65)	<0.001
Female	202 169 (52%)	324 (20%)	<0.001	54 293 (57%)	153 (26%)		43 727 (55%)	28 (15%)	
Male	185 411 (48%)	1276 (80%)		41 812 (44%)	443 (74%)		36 394 (46%)	162 (85%)	
Ethnic background			0.75			0.74			0.40
White	367 547 (95%)	1526 (95%)		92 803 (97%)	579 (98%)		77 490 (97%)	186 (98%)	
Mixed or non-white	18 915 (5%)	67 (4%)		2968 (3%)	15 (2%)		2413 (3%)	3 (2%)	
Missing	1118 (<1%)	7 (<1%)		334 (<1%)	2 (<1%)		218 (<1%)	1 (<1%)	
BMI (kg/m^2^)			<0.001			<0.001			<0.001
<24.9	127 352 (33%)	259 (16%)		36 815 (38%)	120 (20%)		31 033 (39%)	43 (23%)	
≥25 and <30	165 702 (43%)	617 (39%)		39 488 (41%)	247 (42%)		33 073 (41%)	74 (39%)	
≥30 and <35	64 782 (17%)	466 (29%)		13 736 (14%)	144 (25%)		11 209 (14%)	50 (26%)	
≥35	24 159 (6%)	226 (14%)		4810 (5%)	77 (13%)		3783 (5%)	19 (10%)	
Missing	5585 (2%)	32 (2%)		1256 (1%)	8 (1%)		1023 (1%)	4 (2%)	
Alcohol status			<0.001			0.003			<0.004
Never	14 891 (4%)	94 (6%)		2790 (3%)	27 (5%)		2161 (3%)	6 (3%)	
Previous	12 987 (3%)	156 (10%)		2620 (3%)	48 (8%)		2118 (3%)	15 (8%)	
Current	359 513 (93%)	1349 (84%)		90 614 (94%)	521 (87%)		75 825 (95%)	169 (89%)	
Missing	189 (<1%)	1 (<1%)		81 (<1%)	0		17 (<1%)	0	
Smoking status			<0.001			0.002			<0.002
Never	212 488 (55%)	565 (35%)		54 839 (57%)	237 (40%)		45 656 (57%)	77 (41%)	
Previous	134 923 (35%)	842 (53%)		34 477 (36%)	307 (52%)		28 815 (36%)	101 (53%)	
Current	39 197 (10%)	189 (12%)		6633 (7%)	50 (8%)		5526 (7%)	12 (6%)	
Missing	972 (<1%)	0		156 (<1%)	2 (<1%)		124 (<1%)	0	
Socioeconomic status			0.06			0.87			0.13
Q1	77 446 (20%)	213 (13%)		21 154 (22%)	110 (18%)		17 765 (22%)	27 (14%)	
Q2	77 444 (20%)	272 (17%)		20 338 (21%)	130 (22%)		16 987 (21%)	51 (27%)	
Q3	77 451 (20%)	285 (18%)		19 666 (21%)	121 (20%)		16 380 (20%)	33 (17%)	
Q4	77 404 (20%)	330 (21%)		18 969 (20%)	117 (20%)		15 841 (20%)	50 (26%)	
Q5	77 250 (20%)	497 (31%)		15 868 (17%)	118 (20%)		13 047 (16%)	29 (15%)	
Missing	585 (<1%)	0		110 (<1%)	0		101 (<1%)	0	

Quantile borders for the Townsend score were –6.26, –3.97, –2.84, –1.44, 1.11, 11.00 based on the participants with self-reported PA data.

Participants with poor wear quality and missing calibration coefficients are not included in the table. P values were calculated using the Kolmogorov-Smirnov test for continuous variables and the χ^2^ test for categorical variables. The overlapping group refers to individuals who were included in the self-reported analysis and also had sufficient-quality accelerometer recordings.

BMI, Body Mass Index; HF, heart failure; PA, physical activity.

**Figure 2:**
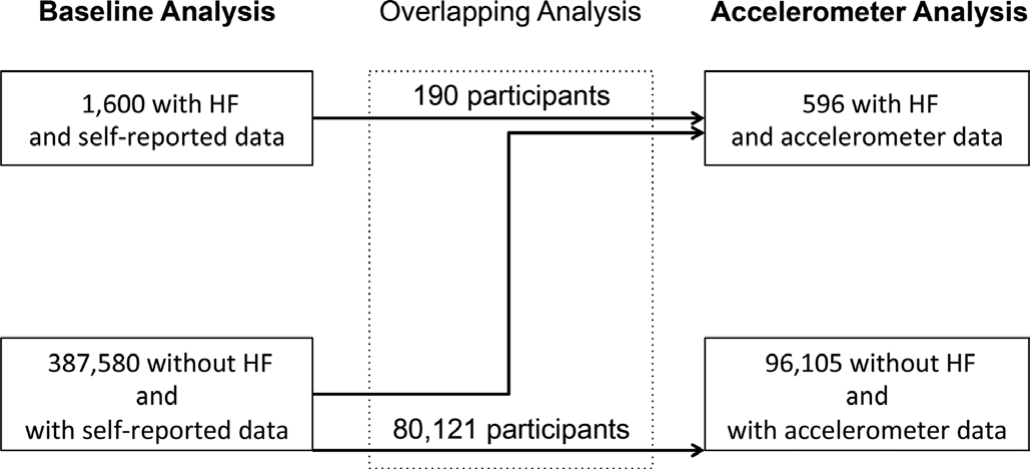
Flowchart of the participants included in the baseline and accelerometer analyses. Some participants included in the accelerometer analysis did not provide self-reported activity data at baseline; others developed HF in the period between baseline assessment and accelerometer recording. Participants falling into both the baseline and accelerometer analysis groups (overlapping analysis) were included in a separate sensitivity analysis. HF, heart failure.

**Figure 3:**
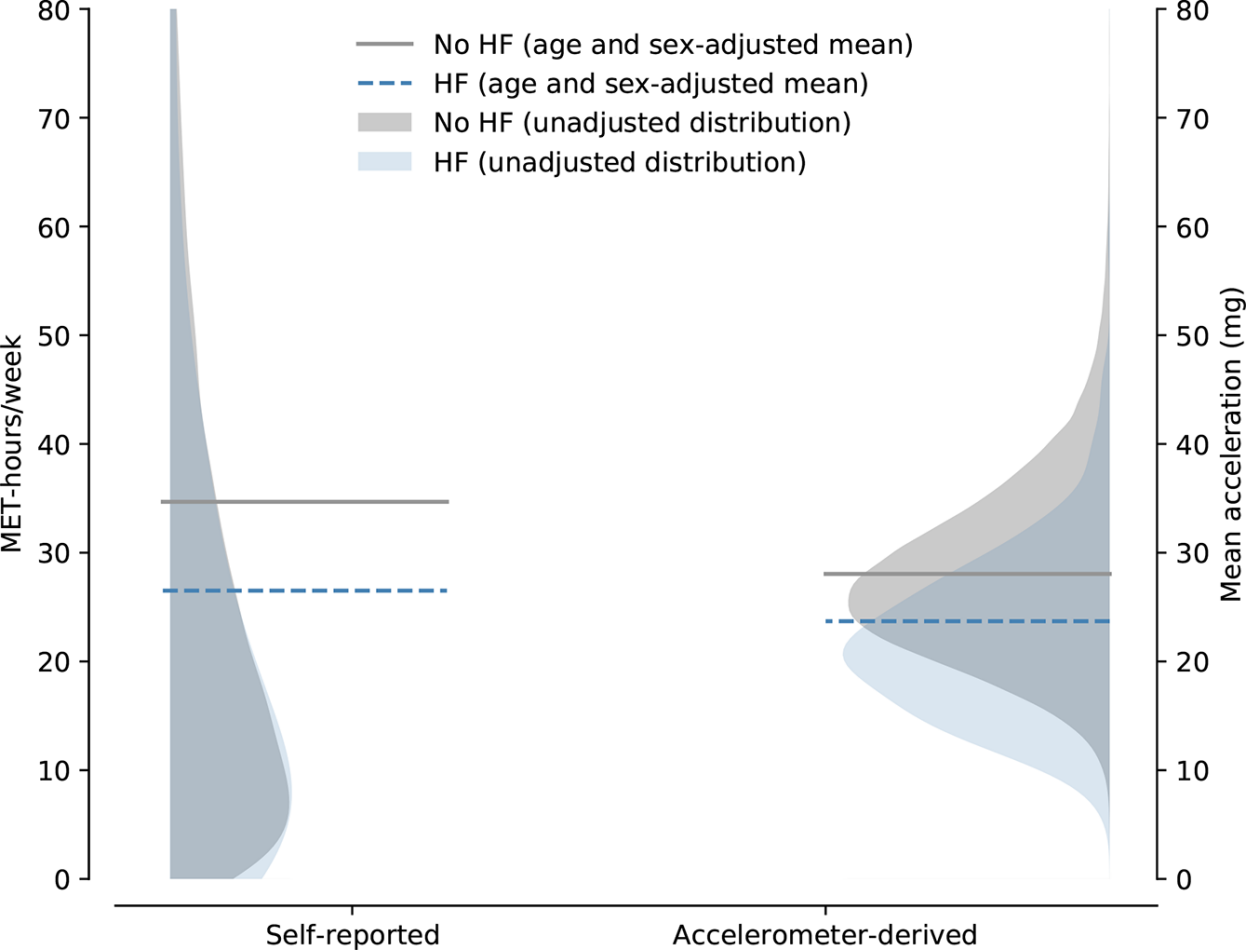
Comparison of self-reported (p<0.001) and accelerometer-derived (p<0.001) physical activityPA in participants with or without heart failureHF. Shaded areas depict unadjusted physical activityPA distributions and dashed lines show age-adjusted/sex-adjusted mean physical activityPA. HF, heart failure; MET, metabolic equivalent of task; PA, physical activity.

The mean age/sex-adjusted self-reported PA level per week was 26.5 (95% CI 24.7 to 28.4) MET-hours per week for participants with diagnosed HF vs 34.7 (95% CI 34.5 to 34.9) MET-hours per week for participants without any prior diagnosis of HF (p<0.01) (see figure 2). The results did not change substantially when the model was further adjusted for BMI, alcohol consumption, smoking status and social deprivation score (table 2). Both times since first and last HF-related hospitalisation did not significantly affect PA levels reported by patients with HF either (table 3). According to the self-reported data, patients with HF spent less time in all types of reported PA, including walking and moderate and vigorous PAs (figure 3A).

**Table 2:** Comparison of mean daily PA in patients with versus without HF with cumulative adjustments for covariates

Model	HF mean PA (CI)	No HF mean PA (CI)	P value of HF status
Self-reported (in METs)			
Baseline (age and sex, HF)	26.5 (24.7 to 28.4)	34.7 (34.5 to 34.9)	<0.001
Baseline+ethnicity	26.6 (24.7 to 28.4)	34.7 (34.5 to 34.9)	<0.001
Baseline+BMI	28.2 (26.4 to 30.1)	34.7 (34.5 to 34.9)	<0.001
Baseline+alcohol status	26.5 (24.6 to 28.3)	34.7 (34.5 to 34.9)	<0.001
Baseline+smoking status	26.4 (24.5 to 28.2)	34.7 (34.5 to 34.9)	<0.001
Baseline+Townsend	26.1 (24.3 to 28.0)	34.7 (34.5 to 34.9)	<0.001
Baseline+all of the above	27.7 (25.8 to 30.0)	34.7 (34.4 to 35.0)	<0.001
Accelerometer-derived (in milligravity)			
Baseline (age and sex, HF)	23.7 (23.1 to 24.4)	28.1 (28.0 to 28.1)	<0.001
Baseline+ethnicity	23.7 (23.1 to 24.4)	28.1 (28.0 to 28.2)	<0.001
Baseline+BMI	24.7 (24.0 to 25.3)	28.1 (28.0 to 28.2)	<0.001
Baseline+alcohol status	23.7 (23.1 to 24.4)	28.1 (28.0 to 28.1)	<0.001
Baseline +smoking status	23.7 (23.1 to 24.4)	28.1 (28.0 to 28.2)	<0.001
Baseline+Townsend	23.8 (23.1 to 24.4)	28.1 (28.0 to 28.2)	<0.001
Baseline+all of the above	24.7 (24.1 to 25.4)	28.1 (27.9 to 28.2)	<0.001

P values are reported for HF status adjusted for the listed covariates.

BMI, Body Mass Index; HF, heart failure; MET, metabolic equivalent of task; PA, physical activity.

**Table 3:** Secondary analysis: adjusting for time since first and last hospitalisation

Model	P value of ‘time since’ variable
Self-reported	
Baseline+time since first diagnosis	0.29
My Baseline+time since last hospitalisation	0.65
Baseline+time since last primary hospitalisation	0.91
Accelerometer-derived	
Within HF	
Baseline+time since first diagnosis	0.69
Baseline+time since last hospitalisation	0.10
Baseline+time since last primary hospitalisation	0.58

The baseline model includes age, sex and HF status as input variables.

HF, heart failure.

During the assessment, participants with diagnosed HF wore their accelerometers for a median of 6.9 (25th and 75th percentiles 6.8 and 7.0, respectively). Participants without HF wore the devices for an average of 6.9 (6.7 and 7.0) days. The mean acceleration values were significantly higher in participants without HF than those with HF (28.1 (95% CI 28.0 to 28.1) milligravity vs 23.7 (95% CI 23.1 to 24.4) milligravity, respectively; p<0.001) (figure 2). This result did not change when additional potential covariates were adjusted for (table 2).

The age/sex-adjusted analysis of the percentage time spent within certain PA intensities showed that patients with HF spent a larger proportion of time sedentary and less time in light, moderate and vigorous PAs (figure 3b). The analysis of hourly mean acceleration values showed that patients with HF were significantly less active throughout the entire day (figure 3c). Between the hours of 06:00 and 19:00, participants without HF achieved approximately 20% higher mean acceleration per hour than HF participants when adjusting for age and sex.

Median times since first HF-related hospitalisation were 3.6 (95% CI 1.6 to 6.1) years for the self-reported analysis and 4.2 (95% CI 2.0 to 8.2) years for the accelerometer analysis. Median times since last hospitalisation were 2.9 (95% CI 1.2 to 5.5) years and 3.3 (95% CI 1.3 to 6.9) years, respectively. The time since first report of HF or since last HF-related hospitalisation did not have a significant impact on the mean acceleration values measured (table 3). The results of the sensitivity analysis, restricting the comparisons to the overlapping group of participants who had HF at baseline and adjusting for age and sex, were broadly consistent with our main results (online supplementary figure S1).

10.1136/openhrt-2019-001099.supp1 Supplementary data

## Discussion

Our analysis of PA levels in the UK Biobank population showed that both perceived and objectively measured PA levels of patients with HF were significantly reduced in comparison with people without HF.

The accelerometer-derived PA comparison showed that individuals with HF were on average 16% less physically active than individuals without a prior diagnosis of HF. This is in agreement with, although slightly lower than, previous findings from small-scale studies, which suggested that patients with HF are 44%–65% less physically active (based on step counts/movement-related activity) than individuals without HF.^9 13 23 24^ Moreover, our study extends previous studies and shows that the average lower-level of PA was not due to a reduction in more intense types of activities but due to a generally less active lifestyle throughout the day, with peak differences occurring between 06:00 and 20:00. To our knowledge, this is the first study giving an insight into HF PA levels throughout the day.

Past studies have shown that self-reported PA levels are low to moderately correlated with objectively recorded PA levels^25 26^ and may be affected by socioeconomic factors.^27^ Despite this, the general message of both the self-reported (perceived) and accelerometer-derived (objective) measurements investigated as part of this analysis was consistent. However, accelerometer recordings can provide a more detailed and precise assessment of activity levels of patients with HF throughout the day and over time.

PA levels of patients with HF within this cohort were not significantly affected by time after diagnosis of HF. These results contrast with findings by Miura *et al*,^2^ who studied self-reported PA levels in 4500 Japanese patients with HF and found that less than 30% of patients with HF managed to maintain their self-reported PA levels over a 1.4year time span. Both selection and survival bias intrinsic to the UK Biobank accelerometer data might have distorted the measured effect of time since first and last HF-related hospitalisations on PA levels. Further studies will therefore be needed to quantify the degree and determinants of change in PA over time.

Limitations of this study include its cross-sectional nature and the fact that both the self-reported and accelerometer data provided by the UK Biobank might be affected by selection bias, resulting in a healthier than normal study population represented in this analysis.^28^ This would lead to a sample not representative of the HF population at large. Thus, our findings may be more applicable to patients with less severe chronic HF.

In summary, our results show that perceived and objectively recorded PA levels of patients with chronic HF are significantly lower than those of individuals without HF. This difference is continuous throughout the different hours of the day and suggests that increases in everyday activity may be a potential alternative to structured exercise programmes.
