# Supplementary figures and images for: Self-reported and objectively measured physical activity in people with and without chronic heart failure: UK Biobank analysis
Source: Open Heart. 2020 Feb 19;7(1):e001099. doi: 10.1136/openhrt-2019-001099 (PMC7046950; doi:10.1136/openhrt-2019-001099)

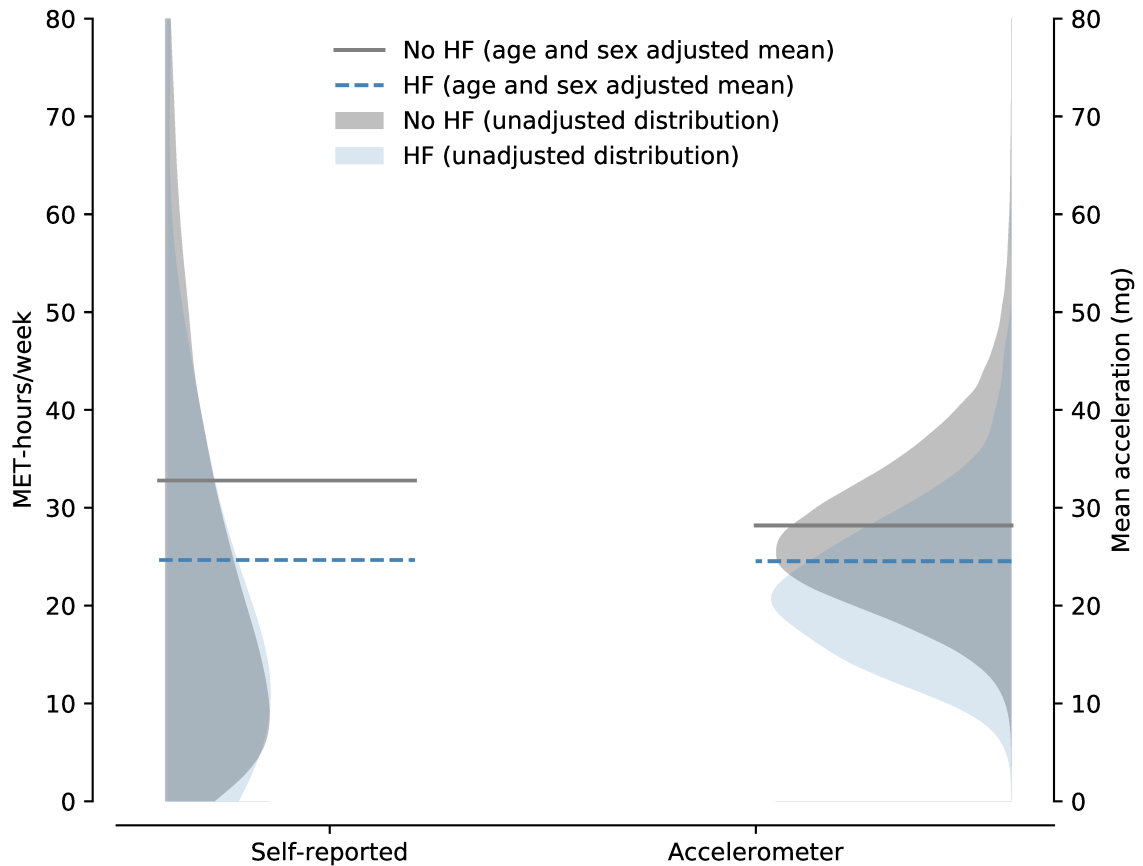

Supplement: Supplementary data [file openhrt-2019-001099supp001.pdf]
